# Ketamine abuse also affects sexual health

**DOI:** 10.1093/sexmed/qfag049

**Published:** 2026-07-22

**Authors:** Eeke C M Leerssen, Wouter M H van der Sanden, Vincent F de Kemp, Rob A Schipper, Michel I A Wyndaele, Laetitia M O Kort

**Affiliations:** Jeroen Bosch Ziekenhuis, GZ ‘s-Hertogenbosch, 5223, The Netherlands; Jeroen Bosch Ziekenhuis, GZ ‘s-Hertogenbosch, 5223, The Netherlands; UMC Utrecht, Utrecht, CX 3584, The Netherlands; Jeroen Bosch Ziekenhuis, GZ ‘s-Hertogenbosch, 5223, The Netherlands; UMC Utrecht, Utrecht, CX 3584, The Netherlands; UMC Utrecht, Utrecht, CX 3584, The Netherlands

**Keywords:** ketamine, ketamine induced uropathy (kiu), quality of life (qol), sexual health, lower urinary tract symptoms (luts), erectile dysfunction

## Abstract

**Introduction:**

Recreational ketamine use is rapidly increasing, and ketamine-induced uropathy (KIU) is gaining recognition for its severe impact on quality of life (QoL), including pain, hematuria, lower urinary tract symptoms (LUTS), and potential negative effects on sexual function. This study investigates the prevalence of sexual dysfunction among men with KIU.

**Methods:**

Male patients with KIU completed the International Index of Erectile Function (IIEF-5 and/or IIEF-15). Erectile dysfunction (ED) was defined as a score of ≤21 on IIEF-5 or ≤ 25 on domain A of IIEF-15. Patients who had not been sexually active in the previous four weeks (a score of 0 on IIEF-5 or within any domain of IIEF-15) were excluded. The impact of LUTS on sexual life and its perceived severity were assessed using items 10a and 10b of the International Consultation on Incontinence Questionnaire LUTS Quality of Life module (ICIQ-LUTSqol). A score > 5 on item 10b was considered indicative of a (major) problem.

**Results:**

A total of 123 men with KIU, mean age 28 years (range: 18-50 years), completed the questionnaire(s): 81 (66%) completed IIEF-5, and 88 (72%) IIEF-15. Only 42% (*N* = 37) had been sexually active in the preceding four weeks. Mean IIEF-5 score in these patients was 18 (SD 5.7), with 63% meeting the criteria for ED. Mean IIEF-15 domain A score was 50 (SD 16.3), with 51% classified as having ED. Beyond ED, 39% reported reduced or absent ejaculation volume, and 52% reported mild to severe pain during ejaculation. Overall, 58-75% reported that LUTS had at least some negative impact on their sexual life, and 64% perceived this impact as a (major) problem.

**Discussion:**

Less than half of men with KIU were sexually active in the past four weeks, with the majority reporting sexual health issues, including frequent erectile dysfunction and ejaculatory dysfunction, which were often perceived as significant problems. Awareness is needed among current and potential recreational ketamine users, as well as their healthcare providers, regarding the detrimental effects of ketamine not only on bladder function but also on sexual health.

## Introduction

Ketamine emerged in the 1960s as a safer anesthetic alternative to phencyclidine. It combined strong analgesic effects with a more favorable safety profile, revolutionizing anesthetic practice.^1^ In addition to its analgesic and anesthetic effects, ketamine also possesses psychotropic properties; it induces hallucinations, altered perceptions and a sense of detachment from reality, described as a “k-hole”, when taken in high doses. These effects have made ketamine popular as a recreational drug since the 1990s.

In recent years, its use in nightlife settings has been on the rise in Europe. This is illustrated by increased levels of ketamine residues in wastewater analysis from several European cities.^2^ Data from the Netherlands indicate that 0.8% of adults used ketamine in 2022, equating to approximately 120 000 individuals. In 2023, nearly a quarter (24.6%) of young adults (16-35 years) who participate in nightlife activities reported using ketamine, which was a slight increase from 2020 (22.1%).^3^ This growing trend is mirrored by a sharp increase in ketamine-related inquiries to the Dutch Poisons Information Centre since 2018, with 139 phone calls and 391 website consultations in 2024 alone.

Ketamine has been associated with potentially devastating urological manifestations: ketamine-induced uropathy (KIU). A wide range of *lower urinary tract symptoms* (LUTS) has been described in KIU, such as urgency, frequency, and dysuria, as well as suprapubic pain and hematuria.^4^ It is known that KIU severely compromises *quality of life* (QoL). Parallel to the rise in recreational ketamine use, the incidence of KIU has increased in recent years. In 2024, 137 patients with KIU were treated at a dedicated ketamine urology outpatient clinic in the Netherlands, up from only 17 in 2022.^5^

In addition to these commonly recognized LUTS, there is an emerging concern regarding the potential influence of ketamine abuse on sexual function in both males and females.^6^ Erectile dysfunction (ED) in males and pain-associated sexual intercourse in females were commonly observed among individuals who abuse ketamine.^7–9^ At present, the relationship between ketamine abuse, KIU and sexual (dys)function in men has not been thoroughly investigated, particularly when assessing a broader range of sexual functions beyond ED. Therefore, the objective of this study is to investigate the prevalence and impact of sexual complaints among men with KIU.

## Materials and methods

A cross-sectional design was used in this study. Men who presented at a dedicated Dutch ketamine urology outpatient clinic between February 2024 and January 2025 with symptoms consistent with KIU, either self-reported or clinically diagnosed, were eligible for inclusion. KIU was defined based on a combination of^1^ a history of recreational ketamine use,^2^ the presence of LUTS (eg, urgency, frequency, dysuria, pain), and^3^ clinical assessment by an expert urologist. In all cases, symptoms were considered in the context of ketamine exposure, with onset or worsening of LUTS during or after periods of ketamine use. All participants received a verbal explanation of the study and provided informed consent for inclusion in a registry for prospective research.

Participants were questioned on their ketamine use at first presentation, including the age at which they first used ketamine, the duration of intensive use (defined as daily use) and the dosage during intensive use (grams per week). For those respondents who had ceased using ketamine for longer than one month, the period of abstinence was recorded (months). These data were collected in conjunction with demographic information and details on the consumption of alcohol and other drugs.

As per standard practice, participants were invited to complete the self-administered *International Consultation on Incontinence Questionnaire Male Sexual Matters Associated with Lower Urinary Tract Symptoms* (ICIQ-MLUTSsex), as well as the *International Consultation on Incontinence Questionnaire LUTS QoL* (ICIQ-LUTSqol*)*. The overall score of the 4 questions of ICIQ-MLUTSsex ranges from 0 to 12 with higher scores indicating increased sexual problems. Items 10a and 10b of ICIQ-LUTSqol relate to the patient’s sex life; a score > 5 on item 10b (bother scale 0-10) was defined as indicating a (major) problem. Patients who selected “not applicable” for item 10a (“Does your urinary problem affect your sex life?”) were excluded from the analysis of item 10b.^9^^,^^10^

Participants also completed the International Index of Erectile Function 5 (IIEF-5) and/or its comprehensive version (IIEF-15), with the IIEF-5 items derived from the IIEF-15 when applicable. A score of 0-5 is awarded to each of the 15 questions that examine five main domains of male sexual function: erectile function (EF), orgasmic function (OF), sexual desire (SD), intercourse satisfaction (IS) and overall satisfaction (OS). Lower scores reflect more severe symptoms. Patients who had not been sexually active during the past four weeks (a score of 0 on IIEF-5 or within one of the domains of IIEF-15) were excluded from further analysis. ED was defined as a score of ≤21 on IIEF-5 or ≤ 25 on Domain A of IIEF-15, respectively. ED within IIEF-15 was assessed only in men who completed all items within domain A. There is no consensus on the cutoff points for normal values in the other domains.^11^

### Statistical analysis

IBM SPSS Statistics for Windows, Version 27.0, was used for analysis. Descriptive statistics were used for demographic data and questionnaire scores. Comparative statistical analyses were performed to evaluate differences in sexual function and related outcomes between subgroups. Independent-samples t-tests were applied for normally distributed continuous variables, while non-parametric equivalents (Mann–Whitney U test) were used when distributional assumptions were not met. Correlations between variables were assessed using Spearman’s rank correlation coefficient (ρ), due to the non-normal distribution and ordinal nature of the data.

All statistical tests were two-tailed, and *P* < .05 was considered statistically significant.

## Results

Between February 2024 and January 2025, a total of 123 men with KIU attending the dedicated ketamine outpatient urology clinic were included in the registry. Mean age was 28 years (range: 18-50 years). Table 1 presents baseline characteristics.

**Table 1:** Baseline characteristics of study participants.

	N (%)/M ± SD
Patients	123 (100%)
Age (years)	28 **±** 6.3
Age at first use (years)	21 **±** 6.0
Clean	48 (39%)
Total duration of intensive ketamine use (months)	45 **±** 36.4
Dosage during intensive use (grams/week)	24.2 ± 18.5
Alcohol use	66 (53.7%)
Cigarette smoking	97 (78.9%)
Use of other drugs	67 (54.5%)

Abbreviations: Clean = abstinent from ketamine use for more than one month, Intensive use = daily ketamine consumption, N = number, M = mean, SD = standard deviation

### I‌IEF-5 and IIEF-15

IIEF-5 was completed by 81 (66%) men, while IIEF-15 was completed by 88 (72%) men. Only 37 (42%) respondents had been sexually active in the preceding four weeks.

Mean IIEF-5 score among sexually active respondents was 18 (SD 5.7), which falls within the range of mild ED. Overall, 63% of men met the criteria for ED (Table 2). No significant difference was found in mean IIEF-5 score between men who were currently clean and those who were not, with scores of 20 (SD 5.4) and 17 (SD 5.6), respectively (*P* = .070).

**Table 2:** IIEF-5 and IIEF-15 mean scores and prevalence of erectile dysfunction.

	M ± SD/N (%)
IIEF-5 *(score 0-25) N = 40*	18 ± 5.7
❖ Erectile dysfunction	25 (63%)
IIEF-15 *(score 0-75) N = 37*	50 ± 16.3
Erectile function *(score 0-30) N = 39*	22 ± 7.4
❖ Erectile dysfunction	20 (51%)
Orgasmic function *(score 0-10) N = 60*	7 ± 2.9
Sexual desire *(score 0-10) N = 87*	6 ± 2.3
Intercourse satisfaction *(score 0-15) N = 40*	10 ± 3.4
Overall satisfaction *(score 0-10) N = 69*	6 ± 2.8

Abbreviations: IIEF = International Index of Erectile Function, M = mean, SD = standard deviation, N = number

Due to incomplete responses, the number of respondents per domain varied.

The average total score on IIEF-15 was 50 (SD 16.3), and 51% of respondents were classified as having ED. Similarly, no significant difference in IIEF-15 total scores was observed between clean and non-clean respondents, with mean scores of 57 (SD 13.7) and 46 (SD 16.6), respectively (*P* = .085).

### ICIQ-MLUTSsex

ICIQ-MLUTSsex was completed by 108 men (88%) and ICIQ-LUTSqol by 106 men (86%).

In addition to symptoms of ED, a substantial proportion of men also reported other sexual health issues. Specifically, 39% of respondents reported reduced or absent ejaculatory volume, and 52% reported experiencing slight to severe pain during ejaculation (Table 3). No significant difference in ejaculatory volume and pain during ejaculation was found between clean and non-clean respondents (*P* = .785 and *P* = .429, respectively). Moreover, LUTS had a significant impact on sexual wellbeing; between 58% and 75% of men with KIU reported that LUTS negatively affected their sex life to some degree, ranging from a little to a lot. Of these, 64% perceived this impact as a moderate to major problem.

**Table 3:** ICIQ-MLUTSsex and ICIQ-LUTSqol questionnaire scores, assessing the impact of LUTS on sexual function and quality of life.

ICIQ-MLUTSsex	M ± SD/N (%)
Do you get erections? (N = 108)	
Yes, with normal rigidity Yes, with reduced rigidity Yes, with severely reduced rigidity No, erection not possible	72 (66.7%) 24 (22.2%) 8 (7.4%) 4 (3.8%)
❖ How much of a problem is this for you? (0-10)	3.0 ± 3.7
Do you have an ejaculation of semen? (N = 107)	
Yes, normal quantity Yes, reduced quantity Yes, significantly reduced quantity No ejaculation	65 (60.1%) 29 (26.8%) 8 (7.4%) 5 (4.6%)
❖ How much of a problem is this for you? (0-10)	2.5 ± 3.4
Do you have pain or discomfort during ejaculation? (N = 104)	
No Yes, slight pain/discomfort Yes, moderate pain/discomfort Yes, severe pain/discomfort	48 (44.4%) 44 (40.7%) 9 (8.3%) 3 (2.8%)
❖ How much of a problem is this for you? (0-10)	3.6 ± 3.6
To what extent do you feel that your sex life has been spoilt by your urinary symptoms? (N = 106)	
Not at all A little Somewhat A lot	27 (25.0%) 33 (30.6%) 22 (20.4%) 24 (22.2%)
❖ How much of a problem is this for you? (0-10)	5.1 ± 3.8
ICIQ-LUTSqol (N = 106)	
10a. Does your urinary problem affect your sex life?	
not applicable not at all slightly moderately a lot	29 (27.4%) 16 (15.1%) 22 (20.8%) 13 (12.3%) 26 (24.5%)
10B. How much does this bother you (score > 5, 0-10)	43.4 (64%)

Abbreviations: ICIQ-MLUTSsex = International Consultation on Incontinence Questionnaire Male Sexual Matters Associated with Lower Urinary Tract Symptoms, ICIQ-LUTSqol = International Consultation on Incontinence Questionnaire Lower Urinary Tract Symptoms Quality of Life Module, M = mean, SD = standard deviation, N = number

Due to incomplete responses, the number of respondents varied slightly across domains.

Additionally, we found no statistically significant correlations between ketamine use characteristics and LUTS-related quality of life (Spearman’s ρ ranging from 0.033 to 0.183; all *P* > .05; see Supplementary Table 1).

## Discussion

This study demonstrates that sexual dysfunction is highly prevalent among young men with KIU, affecting multiple domains of sexual health. Less than half of the respondents had been sexually active in the past four weeks, and among those who were, a substantial majority reported significant sexual complaints. ED was common, with 51%-63% of respondents meeting diagnostic criteria depending on the assessment tool used. However, disturbances in other domains were also frequently observed: over one third reported reduced or absent ejaculatory volume, and more than half experienced pain during ejaculation. Moreover, patients reported that LUTS had a substantial negative impact on their sexual life, with 58%-75% indicating LUTS-related sexual impairment, and 64% of these perceiving this impact as a moderate to severe problem. These results highlight that the consequences of ketamine abuse extend beyond LUTS and that sexual dysfunction is prevalent in men with KIU, and not limited to ED. It constitutes a broader, functional disabling syndrome with substantial effects on QoL.

These findings contrast with what is known in the general young male population. A multinational study from Rosen et al., involving 27 839 men in eight countries, found a prevalence of ED between 8%-11% in men in their 20s and 30s.^12^ Similarly, a Swiss study from Mialon et al., in over 9000 conscripts aged 18-25 years, found that 30% of sexually active men met criteria for ED (IIEF-5 < 22).^13^ In the United States based Growing Up Today Study (GUTS), moderate to severe ED (IIEF-5 ≤ 16) was reported in only 9%-14% of young men aged 18-31 years.^14^ Compared to these cohorts, the prevalence of ED in our KIU population is markedly higher.

The prevalence rates of sexual dysfunction associated with ketamine abuse varied in previous studies.^6^ Yang et al. examined 1056 male ketamine users in Taiwan (mean age 27.4), including 993 street users apprehended by police, and 63 urology patients. ED was present in 30.8% (IIEF-5 ≤ 21), and they found that ED and LUTS were significantly more prevalent among hospital patients than street users (77.6% vs. 6.7%, respectively).^7^ Suppiah et al. surveyed 127 male ketamine and polydrug users in Malaysia (mean age 28.3) and found a 52% ED prevalence based on a single self-reported question. Both studies identified longer ketamine use as a stronger predictor of ED than dosage, suggesting duration of ketamine abuse plays a greater role than dose in sexual dysfunction development. Although differences in populations and ED assessment methods limit direct comparisons, an important distinction is that our cohort consisted exclusively of men with clinically relevant KIU, likely representing a more severe subgroup of ketamine users. In contrast, previous studies often included broader populations of recreational users or relied on less comprehensive measures of sexual function. This may partly explain the higher prevalence of ED observed in our study.

The exact cause of ED in ketamine users remains unclear. It may result from a direct neurotoxic effect of ketamine, as scientific literature shows that repeated high-dose ketamine exposure in young animals induces dose- and time-dependent apoptotic neurodegeneration via multiple molecular pathways. These mechanisms could affect peripheral nerves, potentially contributing to ED. However, evidence for comparable neurotoxicity in humans is limited and inconclusive.^15–17^ Alternatively, ED may be secondary to LUTS and associated pain experienced by these patients. While the etiological basis of KIU is distinct, there appears to be clinical overlap with the pathophysiological mechanisms described in bladder pain syndrome/interstitial cystitis (BPS/IC). Ongoing urothelial injury and local inflammation can lead to peripheral and central sensitization of pelvic afferent pathways. This may result in pain during sexual activity and ejaculation, as well as reduced sexual desire. Chronic bladder inflammation has been shown to affect sexual function in men: 71% of men with BPS/IC report pain associated with sexual activity.^18^ In our cohort of male patients with KIU, 52% reported pain during intercourse.

This study’s strengths are the clearly defined population of men with KIU and the relatively large sample size. Previous studies have either included smaller samples or more heterogeneous populations of ketamine users. Using multiple validated instruments, we assessed a broader than before spectrum of sexual functions, including erectile, ejaculatory, and orgasmic, as well as sexual satisfaction. Although both IIEF-5 and IIEF-15 were used, derivation of IIEF-5 scores from IIEF-15 responses ensured consistency in the assessment of erectile function across participants. The sexual function complaints in male patients with KIU are relatively common in this group but remain underrecognized and insufficiently studied. Due to the concomitant evaluation of the perceived impact of LUTS on sexual life, this study offers a more comprehensive understanding of the sexual health burden in men with KIU and underscores the need for greater clinical awareness and further research.

A possible limitation of the study is the restriction of IIEF questionnaires to only assess sexual function within the past four weeks, thereby excluding patients who were not sexually active in that timeframe. This may reduce the generalizability of our results. Patients experiencing sexual dysfunction, or painful LUTS such as in KIU, for a longer time may eventually refrain from sexual activity and be missed in our assessment during the “past four weeks” window. The prevalence of sexual inactivity in this young male population is surprisingly high, and may indicate an even higher occurrence of sexual dysfunction than found in our study. Additionally, IIEF offers only a limited assessment of the broader psychosexual context. It does not adequately account for factors such as partner dynamics, relational stability, or sexual orientation, nor does it distinguish between different forms of sexual activity (eg, intercourse versus masturbation).^18^ These nuances are particularly relevant in younger individuals with substance use disorders, as trauma- and stressor-related disorders frequently co-occur in this population, all of which may independently influence sexual function.^19^

On the other hand, the ICIQ-MLUTSsex questionnaire does not exclude sexually inactive patients. Responses may be subject to recall bias which may further complicate interpretation of responses among sexually inactive individuals.^20^ Other limitations are inherent to the cross-sectional design of this study. Causal inference is limited, as temporal relationships between ketamine use and sexual dysfunction cannot be assessed, and neither can change over time. Selection bias may affect the sample due to the self-selected nature of participation. Furthermore, urine toxicology screening was not conducted; although self-reported abstinence was accepted, some degree of misclassification cannot be ruled out in this population. Such reliance on self-report is a well-recognized limitation in addiction research and is not unique to the present study. Lastly, we did not analyze the combined use of alcohol, cigarettes, and other drugs, while the high prevalence of polydrug use in this cohort may have further contributed to sexual dysfunction.

This study highlights the high prevalence of sexual dysfunction among men with KIU, yet this issue has been underrecognized in clinical practice and public health to date. There is a clear need to raise awareness among recreational ketamine users and healthcare providers about the broad range of sexual health problems, beyond LUT dysfunction, including erectile and ejaculatory disorders. Given the multidisciplinary relevance, clinicians in urology, psychiatry, addiction medicine, and sexual health should proactively address sexual health in young or ketamine-using patients, to enable early detection and reduce stigma.

Finally, these findings emphasize the need for longitudinal research to assess sexual function recovery after ketamine abstinence and to clarify the underlying mechanisms, allowing development of future interventions. Future research should also explore sexual dysfunction in women and potential effects on male fertility.

## Conclusion

Less than half of the men with KIU had been sexually active in the past four weeks. Most of them reported significant sexual health issues. Not only erectile but also ejaculatory dysfunction was common and often perceived as a problem. Awareness should be raised among both (potential) ketamine users and healthcare professionals, who should proactively address sexual health in their evaluation of patients with KIU.

## Supplementary Material

Supplementary_material_qfag049
