# Supplementary material for: Ketamine abuse also affects sexual health
Source: Sex Med. 2026 Jul 22;14(5):qfag049. doi: 10.1093/sexmed/qfag049 (PMC13390908; doi:10.1093/sexmed/qfag049)
Supplement: Supplementary_material_qfag049 [file supplementary_material_qfag049.docx]

Supplementary Table 1: Ketamine use characteristics and LUTS-related quality of life.

|  | Spearman’s ρ | p-value | N |
| --- | --- | --- | --- |
| Duration of intensive ketamine use (months) | 0.033 | 0.730 | 110 |
| Dosage during intensive use (g/week) | 0.183 | 0.051 | 114 |
| Months of abstinence | 0.060 | 0.523 | 116 |
